# Torsion d'annexe au second trimestre de la grossesse, à propos de deux cas

**DOI:** 10.11604/pamj.2016.25.113.9170

**Published:** 2016-10-25

**Authors:** Amira Ayachi, Zeineb Blel, Nahed Khelifa, Lassaad Mkaouer, Rim Bouchahda, Mechaal Mourali

**Affiliations:** 1 Université El Manar 2, Tunis, Tunisie; 2 Faculté de Médecine de Tunis, Tunisie; 3 Service de Gynécologie et Obstétrique, CHU Bougatfa, Bizerte, Tunisie

**Keywords:** Torsion d´annexe, deuxième trimestre, grossesse, Adnexal torsion, second trimester, pregnancy

## Abstract

Les douleurs pelviennes aigues pendant la grossesse peuvent poser un problème de diagnostic différentiel.Nous rapportons deux cas de torsion d'annexes au deuxième trimestre de la grossesse afin d'attirer l'attention sur ce diagnostic, dont seule une prise en charge précoce permet d'éviter des lésions irréversibles dues à l'ischémie, pouvant mettre en jeu le pronostic ultérieur de fertilité. La première patiente, G1P0, enceinte à 20 SA, s'est présenté initialement pour un syndrome appendiculaire. Une incision de Mac Burney, au cours de l'exploration, a montré un ovaire droit nécrosé et une ovariectomie a été faite. Les suites post opératoires étaient simples. La seconde patiente, G2P2, s'est présenté aux urgences avec des douleurs aigues de la fosse iliaque gauche à 26 SA. La laparotomie a mis en évidence une torsion d'une hydatide de Morgani, dont l'aspect nécrosé dû à une torsion, a orienté vers une ablation de l'hydatide. Pour les deux patientes, aucune complication postopératoire n'a été relevée. La torsion d'annexe est une urgence à ne pas méconnaître devant toute douleur pelvienne aigue chez la femme enceinte. Le traitement conservateur est actuellement le gold standard et une prise en charge appropriée est nécessaire pour éviter d'éventuelles complications maternelles et fœtales.

## Abstract (en)

Acute pelvic pain during pregnancy makes the differential diagnosis more challenging. We here report two cases of adnexal torsion during the second trimester of pregnancy in order to draw attention to this diagnosis and to highlight the importance of early treatment to avoid irreversible damages due to ischemia which can be fertility-threatening. The first patient, G1P0, 20 weeks pregnant, initially presented with appendix syndrome. Exploration with a small McBurney incision showed a right ovarian necrosis, hence ovariectomy was performed. The postoperative course was uneventful. The second patient, G2P2, 26 weeks pregnant, presented to the emergency departments with acute left iliac fossa pain. Laparotomy revealed the torsion of a hydatid of Morgagni whose necrotic appearance due to twisting required hydatid ablation. No postoperative complications were noted in the two patients. Adnexal torsion is an emergency condition that should not be ignored in the case of acute pelvic pain in pregnant women. Conservative treatment represents the gold standard and proper management is necessary to avoid possible maternal and fetal complications.

## Introduction

La torsion d'annexe représente la cinquième urgence chirurgicale en gynécologie. La survenue d'une torsion d'annexe sur ovaire sain est une situation rare. 0,2% des femmes enceintes ont subi au moins une intervention pendant la grossesse. Cependant le diagnostic reste difficile, du fait de l'ascension de l'ovaire dans les grossesses avancées pouvant mimer d'autres urgences chirurgicales notamment l'appendicite aigue, la cholécystite ou une pyélonéphrite aigue. Nous rapportons ici deux cas de torsion d'annexe, sur ovaire sain, au cours de la grossesse au deuxième trimestre. L'intérêt de cette situation réside dans sa difficulté diagnostique, et dans le choix de l'attitude thérapeutique à adopter. En effet, en l'absence de formation kystique ovarienne, quand faut-il évoquer ce diagnostic? Quel est la voie d'abord privilégiée? Quel geste chirurgical doit-on réaliser sur une torsion d'annexe survenue sur un ovaire sain pour éviter sa récidive?

## Patient et observation

### Observation 1

**Figure 1:**
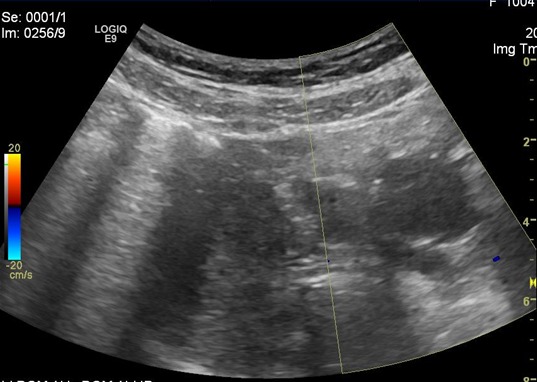
Absence de flux au Doppler couleur au niveau de l'ovaire droit

**Figure 2:**
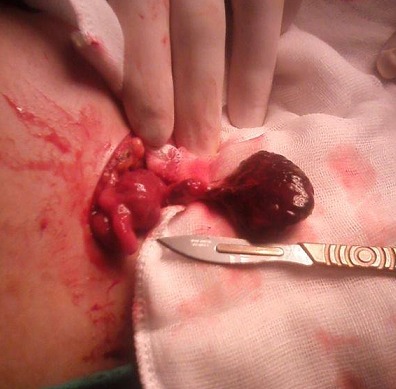
Ovaire nécrosé en per opératoire

Madame H, âgée de 28 ans, consulte pour douleur de la fosse iliaque droite sur une grossesse de 20 SA +3 jours évoluant depuis 6 jours. À l´examen, la patiente était apyrétique, en bon état général. L´examen abdominal trouve une sensibilité de la fosse iliaque droite avec un signe de Rovsing positif. L´examen gynécologique: l´utérus est relâché, au spéculum: le col est macroscopiquement sain, pas de métrorragies ni leucorrhées et au toucher vaginal le col est long fermé postérieur. L´échographie abdominale trouve une grossesse monofoetale évolutive avec une biométrie conforme au terme, un placenta homogène normalement inséré, liquide amniotique en quantité normale, une longueur cervicale à 40 mm. Les deux reins sont bien différenciés, aux contours réguliers, sans lésion apparente. Une distension pyélocalicielle droite de contenu transonore sans obstacle lithiasique décelable. L´appendice est normal. Pas de masse abdominale. L´ovaire droit est superficiel sous la paroi abdominale, de taille normale, non vascularisé au doppler, avec douleur acquise à la pression au passage de la sonde. Présence d´une lame d´épanchement péri-ovarien. L´ovaire gauche est de taille normale folliculaire et bien vascularisé au doppler (Figure 1). La biologie ne trouve ni hyperleucocytose ni ascension de la CRP et un ECBU négatif. Le diagnostic de torsion d´annexe droit a été évoqué ainsi que le diagnostic d´appendicite aigue. La patiente a été opérée sous anesthésie générale, par une incision de Mac Burney. L´exploration trouve un ovaire droit nécrosé tordu avec deux tours de spires, un appendice d´aspect inflammatoire, boudiné (Figure 2). Les gestes effectués sont: une appendicectomie et une ovariectomie droite. Les suites opératoires étaient simples tant sur le plan obstétrical que sur le plan chirurgical avec un bon pronostic d´accouchement par voie basse à terme.

### Observation 2

**Figure 3:**
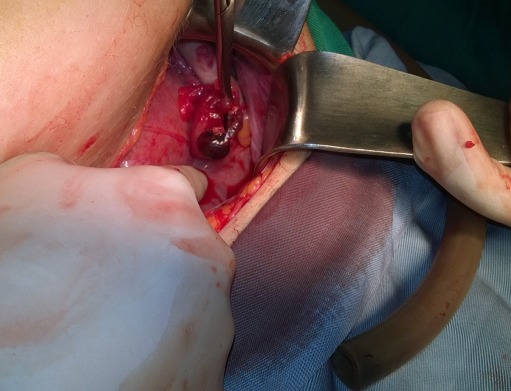
Torsion d'une hydatide sessile avec ovaire sain, grossesse de 27 SA

Mme M.L. âgée de 30 ans, 2^ème^ geste 2^ème^ pare consulte pour douleur fosse iliaque gauche paroxystique à un terme de 26 SA, dans un contexte d'apyrexie. L'examen est sans particularité mis à part une sensibilité au niveau de la fosse iliaque gauche. L'échographie était sans anomalies. Devant la persistance des douleurs, une exploration chirurgicale a été décidée. L'abord a été fait par une incision transversale selon pfannenstiel et l'exploration retrouve au niveau de la trompe gauche une hydatide pédiculée tordue sur son axe, une trompe et un ovaire sain (Figure 3). Une ablation de l'hydatide a été faite. Les suites sont simples et la patientes sortante à J2 post opératoire avec une grossesse évolutive.

## Discussion

Durant la grossesse, la torsion d'annexe est une urgence rare. Son incidence varie de 3 à 5 pour 10000 grossesses [1, 2]. Entre 8 et 28% des torsions surviennent en cours de grossesse [3, 4], majoritairement au premier trimestre mais peuvent être diagnostiquées à tout âge de la grossesse [4]. Habituellement la torsion se produit sur un ovaire pathologique: tumeur maligne ou bénigne, kyste du corps jaune, ou à l'occasion d'un syndrome d' hyperstimulation ovarienne en début de grossesse [3]. Les symptômes se caractérisent par une douleur pelvienne latérale brutale associée à des nausées et des vomissements. Son diagnostic durant la grossesse est rendu complexe car il nécessite l'élimination des diagnostics différentiels classiques mais aussi ceux pouvant être liés à la grossesse (fausse couche, hématome rétro-placentaire, rupture utérine). De plus, l'examen clinique comme les examens d'imagerie deviennent plus difficile en raison du volume utérin et de l'ascension concomitante de l'ovaire dans la cavité abdominale. L'échographie est l'examen de référence. Il permet d'éliminer les diagnostics différentiels, et recherche les facteurs pouvant favoriser la torsion et les signes indirects d'ischémie. L'interruption première du flux veineux entraîne un œdème réactionnel qui est repérable par l´augmentation du volume ovarien comparativement au côté controlatéral [5, 6]. Par ailleurs, l'augmentation du nombre de follicules corticaux est un aspect non spécifique mais qui a été de multiples fois retrouvé dans le cas de torsion sur ovaire sain. Elle serait due à une transsudation liquidienne secondaire à la congestion ovarienne [6, 7]. Cet aspect de structure folliculaire homogène et périphérique a été retrouvé dans notre cas.

Enfin, des images d'infarctus hémorragique peuvent être visualisées tardivement, et une lame d'ascite aspécifique est souvent associée [8]. L'échographie a l'avantage de localiser l´ovaire dans la cavité abdominale et de rechercher une douleur élective au passage de la sonde. L'utilité du doppler des vaisseaux ovariens est controversée. Bien que l'absence de signal doppler confirme l'absence de flux artériel ou veineux et donc la torsion, l´inverse n'est pas vrai [9]. L'IRM est une technique d'exploration complémentaire satisfaisante chez la femme enceinte, qui a le même intérêt que l'échographie avec une plus grande précision [10]. L'association du doppler et de l'IRM est utile mais ne doit pas retarder la prise en charge chirurgicale. Classiquement, la prévention d'une récidive passe dans un premier temps par le traitement étiologique: kystectomie, ponction folliculaire ou annexectomie. Dans le cas d'ovaire sain, la situation est différente. L'étude récente rétrospective de Pansky et al. montre chez des femmes en période post-pubertaire un taux de récidive de 67% lorsque la torsion concerne un ovaire sain (dont 50% du même côté) contre 8% quand l'ovaire est pathologique [6]. L'ovariopéxie bilatérale semble donc être nécessaire [6, 11]. De nombreuses techniques ont été décrites: fixation de l'ovaire au ligament large ou sur paroi latérale, raccourcissement du ligament utéro-ovarien ou fixation de l'ovaire à l'utérus [12–14]. Cette dernière ne semble pas être raisonnablement applicable pendant la grossesse. Deux cas de fixation ovarienne pendant la grossesse seulement ont été rapportés dans la littérature [11, 13]. Djavadjan et al, ont effectué à 15 SA une plicature du ligament utéro-ovarien en suturant la portion la plus proximale de ce ligament à sa portion la plus distale. Lors d'un cas de torsion récidivante sur une grossesse de 10 SA obtenue par FIV, Weitzman et al, décrivent le raccourcissement du ligament utéro-ovarien à l´aide d'un endoloop en raison du risque hémorragique dû à l'hyper-vascularisation. La réalisation de ces techniques pose surtout problème au 2^ème^ et 3^ème^ trimestre du fait de l'augmentation de taille de l´utérus. D'autre part, l´efficacité et l'innocuité de l'ovariopéxie n'ont pas été démontrées. Le raccourcissement du ligament utéro-ovarien pourrait être à l'origine d'une diminution de la vascularisation ovarienne et donc d'une altération de la fonction ovarienne [6]. Nous pouvons imaginer en outre que la fixation latérale pariétale pourrait être à l'origine d'une perturbation anatomique des rapports tubo-ovariens, et d'occlusion sur bride [6]. Ainsi le choix de la technique est laissé à l'opérateur en fonction des contraintes anatomiques spécifiques de chaque patiente. Quelle que soit celle choisie, un traitement préventif controlatéral pourrait éviter les récidives controlatérales au cours de la grossesse. La prise en charge cœlioscopique des pathologies annexielles au premier et au deuxième trimestre de la grossesse n'est plus discutée. Oelsner et al, ont comparé les suites de 197 laparotomies et de 192 laparoscopies sur 17 centres [14]. La cœlioscopie n'augmente pas le risque d'avortement spontané, d'accouchement prématuré, de retard de croissance intra-utérin ou de malformation fœtale par rapport à la laparotomie. Elle présente de plus un risque significativement plus bas de complication postopératoire. Les recommandations du Collège National des Gynécologues et Obstétriciens Français (CNGOF) préconisent la technique cœlioscopique dans la prise en charge des kystes de l'ovaire pendant la grossesse jusqu'à 16-17 SA (niveau de preuve 3), en précisant que la laparotomie est la voie d'abord la plus évaluée après 17SA [15]. Mais plusieurs séries rétrospectives ont montré l'innocuité et l'efficacité de la cœlioscopie au 3ème trimestre [4, 13, 16, 17]. Dans notre cas, nous nous sommes résolus à un abord par laparotomie de type Mac Burney et une incision de Pfannenstiel devant le refus des patientes connaissant les risques encourus pour sa grossesse, risques bien expliqués par deux médecins différents. En peropératoire, devant un ovaire totalement nécrosé et la contigüité avec un appendice inflammatoire, nous avons opté pour une ovariectomie pour la première patiente. Pour la seconde, une ablation de l'hydatide a suffi. Nous avons pris le parti, comme Mathevet et al [16], de tocolyser systématiquement, contrairement à Roman qui ne préconise pas de tocolyse en l'absence de contraction utérine [17].

## Conclusion

Classiquement décrite sur des ovaires ou des trompes pathologiques, la torsion d'annexe peut cependant survenir rarement sur ovaire sain [18]. La récidive en l'absence de traitement préventif est fréquente dans cette situation. L'ovariopéxie bilatérale semble alors être une option thérapeutique raisonnable, que ce soit par ovariopéxie sur la face latérale de la paroi abdominale, ou le raccourcissement du ligament utéro-ovarien. En cours de grossesse cette technique est réalisable par cœlioscopie, cet abord chirurgical devant être privilégié. Toutefois, en fin de deuxième et au troisième trimestre de grossesse, un abord par laparotomie latérale pour un accès direct à l‘annexe peut parfois s´avérer nécessaire.
